# Giant anal condyloma

**DOI:** 10.11604/pamj.2020.35.86.20147

**Published:** 2020-03-24

**Authors:** George Kirkilessis, Ioannis Karavokyros

**Affiliations:** 1 Surgical Department, General Hospital of Pirgos, Pirgos, Greece; 2 First Department of Surgery, Medical School, National and Kapodistrian University of Athens, Laikon General Hospital, Athens, Greece

**Keywords:** Anal condyloma, ulcerated tumor mass, condyloma acuminatum

## Image in medicine

A 61 years old male patient presented to the Accident and Emergency (AE) Department reporting bleeding during defecation, difficulty and low pain while walking and sitting. On physical examination we saw an exophytic cauliflower like, ulcerated tumor mass measuring 20 x 14 cm that covered the whole perineum and a similar satellite lesion 3 x 4 cm, with malodorousness. The mass was there for at least three years, progressively growing.

**Figure 1:**
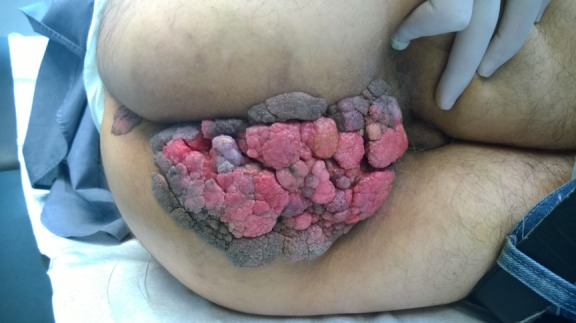
A giant anal condyloma of a 61 years old man with the satellite lesion before the operation

